# Ethical considerations of providers and clients on HIV testing campaigns in Burkina Faso

**DOI:** 10.1186/1472-698X-14-27

**Published:** 2014-10-16

**Authors:** Alice Desclaux, Odette Ky-Zerbo, Jean-François Somé, Carla Makhlouf Obermeyer

**Affiliations:** 1 Institut de Recherche pour le Développement, TransVIHMI UMI 233 (IRD, Université Montpellier 1, Université Cheikh Anta Diop de Dakar, Université de Yaoundé 1), Dakar, Senegal; 2 Programme d’Appui au Monde Associatif et Communautaire (PAMAC), Ouagadougou, Burkina Faso; 3 UNDP, Lomé, Togo; 4 Center for Research on Population and Health, Faculty of Health Sciences, American University of Beirut, Beirut, Lebanon; 5 IRD/CRCF, BP 1386, 18524, Dakar, Senegal

**Keywords:** HIV, Testing campaign, Ethics, Burkina Faso, Individual rights, Global norms, Stigma, Africa

## Abstract

**Background:**

Campaigns have been conducted in a number of low HIV prevalence African settings, as a strategy to expand HIV testing, and it is important to assess the extent to which individual rights and quality of care are protected during campaigns. In this article we investigate provider and client perceptions of ethical issues, including whether they think that accessibility of counseling and testing sites during campaigns may hinder confidentiality.

**Methods:**

To examine how campaigns have functioned in Burkina Faso, we undertook a qualitative study based on individual interviews and focus group discussions with 52 people (providers and clients tested during or outside campaigns and individuals never tested). Thematic analysis was performed on discourse about perceptions and experiences of HIV-testing campaigns, quality of care and individual rights.

**Results:**

Respondents value testing accessibility and attractiveness during campaigns; clients emphasize convenience, ripple effect, the sense of not being alone, and the anonymity resulting from high attendance. Confronted with numerous clients, providers develop context-specific strategies to ensure consent, counseling, confidentiality and retention in the testing process, and they adapt to workplace arrangements, local resources and social norms. Clients appreciate the quality of care during campaigns. However, new ethical issues arise about confidentiality and accessibility. Confidentiality of HIV-status may be jeopardized due to local social norms that encourage people to share their results with others, when HIV-positive people may not wish to do so. Providers’ ethical concerns are consistent with WHO norms known as the ‘5 Cs,’ though articulated differently. Clients and providers value the accessibility of testing for all during campaigns, and consider it an ethical matter. The study yields insights on the way global norms are adapted or negotiated locally.

**Conclusions:**

Future global recommendations for HIV testing and counseling campaigns should consider accessibility and propose ways for testing services to respond to new ethical issues related to high demand.

## Background

Since the beginning of the global HIV/AIDS epidemic, HIV testing has raised a number of issues regarding ethics and human rights. The main concern in the early phases was that HIV tests may be conducted without consent and put people at risk of being stigmatized, especially those already vulnerable [1]. To prevent this, international organizations endorsed informed and voluntary testing and disclosure, requiring three components known as the ‘3 Cs’: Confidentiality, Counseling and informed Consent [2]. These global norms for HIV testing were applied in Sub-Saharan Africa with local particularities regarding interpretations in policies and practices [3]. These requirements were maintained even as the balance of the risks and benefits of HIV testing progressively changed during the early 2000s due to greater availability of antiretrovirals and lower stigma. The PITC (Provider-Initiated Testing and Counseling) strategy introduced in 2007 to expand testing raised concerns that the decision to be tested would shift from the client to the provider. As a result, the ‘3 Cs’ were re-affirmed as a safeguard [4], and efforts to expand testing services were marked by a continuing tension between the drive to diagnose HIV-positive people and the need to protect individual autonomy, privacy and access to care.

Recent strategies to expand HIV testing beyond health facilities have renewed interest in these ethical issues. In 2012, WHO recommended that, in order to reach universal access, rapid testing should be available through a wide range of service-delivery models and approaches, including offering testing in non-medical settings by non-medical personnel, community outreach, door-to-door home-based testing, index patient testing, self-testing and others, and that each country should select the combination of services best adapted to its population [5]. In Southern and East Africa, home-based HIV Counseling and Testing (HIVCT) has been expanded, along with home-based index patient testing and campaigns [5]. In West Africa, low prevalence rates and fear of stigma led to a lower emphasis on home-based testing, and other strategies were developed, including national campaigns to reach general populations and particular subgroups, to motivate individuals and couples, and to mobilize communities to support and encourage testing. Public health concerns led to a new formulation of global norms that better articulated HIVCT within a ‘continuum of care’, and the “3-Cs” became ‘5 Cs’: informed Consent, Confidentiality, Counseling, Correct test results and Connection/linkage to prevention, care and treatment [5].

WHO guidelines emphasize the need to evaluate the ethical aspects of HIVCT strategies, including quality assurance of counseling and testing [6], and increasing attention has been paid to how ethics is embedded in the range of testing practices in a diversity of contexts. An analysis of the indicators of the ‘3 Cs’ in high and low HIV prevalence African countries (Uganda, Kenya, Malawi and Burkina Faso) found favorable outcomes for counseling, consent and confidentiality, along with referral [7]. Provider-initiated ways of delivering HIVCT did not appear to be associated with less favorable outcomes for clients than traditional, client-initiated VCT (voluntary counseling and testing) services [7].

A review of testing policies in these countries found that the question is no longer whether to scale up routine PICT and other strategies, but how it should be scaled up [3]. Evidence about home-based testing indicates high uptake by individuals partly due to greater trust in protection of confidentiality and better accessibility than in health facilities [8]. In this context, couple counseling and testing has a good acceptability and difference in uptake rates according to gender or education level is reduced during follow-up [9]. As reported in Zambia, home-based testing contributed to long-term reduction of stigma [10]. There has been comparatively less attention to campaigns, but a review found that while they increase awareness of potential benefits of the HIV test, campaigns may pressure those who – for whatever reason – do not wish to be tested [11]. It is therefore important to investigate to what extent testing practices meet ethical principles when implemented during campaigns, particularly in low prevalence countries where the level of stigma may be high.

The aim of this article is to analyze provider and client perceptions of ethical issues surrounding HIV-testing campaigns, regarding access, quality of care and individual rights to understand how these play out on the ground. It is based on client and provider experience during HIVCT national campaigns and examines how global norms are adapted by providers in order to protect clients. Special attention is directed to the question of whether reaching high numbers of persons who get tested results in infringements of individual rights to confidentiality, consent and quality of care.

We explored these questions in Burkina Faso, a West African country where HIV prevalence is less than 2% and the epidemic is concentrated in ‘vulnerable sub-populations’ [12]. Campaigns have been an important strategy in Burkina Faso, where HIV-testing rates are considered too low for preventive and treatment strategies to be effective [13].

Community-based organizations in Burkina Faso have played an important role in mobilizing against the epidemic, raising awareness about the need to know one’s HIV status and offering HIV testing to communities. VCT services were started in 1994 by a network of community-based AIDS organizations. VCT facilities were opened in 2001, and the first national awareness campaign for HIV testing was launched in 2003. In 2007, a partnership between the UNDP-supported Programme d’Appui au Monde Associatif et Communautaire (PAMAC, Support Program for Associations and Community Based Organizations) and national AIDS institutions developed a strategy to support VCT centers and mobile strategies in collaboration with community-based organizations. Several annual campaigns were organized, directed at the general population, as well as more targeted campaigns to reach vulnerable or key populations at higher risk [14]. During campaigns, information and awareness interventions defined by national and regional committees are led by community organizations, the media and health care teams [14]. Extra testing sites are open for collective information and individual counseling, and testing is free for those who request it [14]. Rapid tests are used at most sites, which makes it possible to give the results and provide post-test counseling on the same day [14]. National guidelines for counseling and testing do not recommend particular adaptation of counseling modalities and content to the context of campaigns [15,16].

Between 2006 and 2010, campaigns tested 487,727 people, half of the total number of voluntary tests conducted at the national level in community sites and integrated care facilities [17]. One quarter of HIV-positive people tested at national level were diagnosed during campaigns. This achievement is particularly important in a country where only 25% of adults have ever taken an HIV test and 10% tested in the previous 12 months [18]. Evaluations show that campaigns have tested difficult-to-reach populations (especially young people), at a lower cost [17]. The 2008 media awareness campaign whose slogan was ‘I am a leader, I took my test’, involved the participation of traditional authorities and young leaders who took a test and reported it publicly. It resulted in 98,867 people taking an HIV test of which 1866 were diagnosed HIV-positive (1.89%) [17].

Were these good results in terms of numbers of people tested obtained at the expense of individual rights, including autonomy and privacy? Did the campaign raise other ethical concerns? Examining providers’ and clients’ perceptions and experiences about ethical issues during campaigns will help answer these questions and provide key information to evaluate the campaign strategy.

## Methods

The aim of this qualitative study was to bring insights into perceptions of quality of care and ethical issues during HIVCT campaigns by the actors who experience them at field level, in Burkina Faso. Its scope was to provide exploratory data from a variety of perspectives: providers, people who had undergone an HIV test during campaigns, people who had tested outside campaigns, and people who had not been tested. This survey was part of a research program on HIV counseling and testing practices in Africa (Project MATCH: Multi-country African Testing and Counseling for HIV) aimed at exploring public health and ethics issues in four countries (Malawi, Burkina Faso, Kenya, Uganda) based on mixed methods (quantitative survey and qualitative studies) [7,11]. This qualitative sub-study was specific to Burkina Faso. An analysis of campaigns efficiency and costs is presented elsewhere [17].

The study population was composed of 4 sub-populations, defined as follows. *Providers* were people who usually provide HIV counseling along with social or medical care in health centers and community-based facilities. *Clients* were defined as persons we met in the facility at the time of the study, whatever the motivation for their attendance at the service. Amongst them, *Testers* were defined as persons who had an HIV test during their lifetime, and *Non-testers* as those who had not undergone an HIV test. *Testers* had been tested during campaigns (*Campaign testers*) or outside campaigns (*Off-campaign testers*).

We used two main approaches for data collection: focus group discussions (FGDs) with clients (6) and in-depth individual interviews with providers (6). Observation of testing sites (3) was used to triangulate information obtained through FGDs and interviews, and to provide context data. The FGDs included two FGDs held with persons who were never tested, two FGDs held with people who were tested during campaigns and two FGDs held with people tested outside campaigns. Data were collected during the national information and testing campaign in December 2008. The study was conducted in 4 facilities: 2 health services offering PICT and VCT and 2 community-based VCT centers, randomly selected in Ouagadougou (the capital, 1.9 million) and Dedougou (a city of about 100,000 inhabitants), in collaboration with HIV/AIDS community-based organizations, in order to promote trust and facilitate the expression of critical opinions. The research project was cleared by the WHO Ethics Committee and the Ethics Committee for Health Research in Burkina Faso.

Adult clients attending the facility, randomly selected, were invited to participate by health professionals or by members of community-based associations who explained the purpose of the study. Those who agreed were directed to the study interviewers. Focus groups were formed after participants had been informed of the study and had given informed consent, and when a minimum number of six people (and a maximum of ten people) was reached. FGDs were held in a room of the facility, or in one case outside in the facility compound. Individual interviews were conducted with clients anonymously, and they were not asked their HIV-status. Providers were selected from among those highly experienced with HIVCT in each facility and were invited to participate in an interview after working hours. After informed consent, interviews were held in their office or in other suitable rooms. Interviews and FGDs were conducted by four interviewers in two national official languages: Moorè (language of the Moose population, which is also lingua franca for other ethnic groups), Dioula (second most spoken language) and French. Issues covered in the FGDs included perceptions of testing during or out of campaigns, reasons for testing or not-testing, accessibility of testing, knowledge about testing and social consequences of being HIV-positive, personal experience and ethical aspects regarding counseling and testing during and off campaigns. Issues covered in interviews with providers included personal history and experience in counseling and testing, practices and management of testing in their own HIVCT site, difficulties and achievements regarding quality of care and ethics, comparison between campaign settings and routine practice, and suggestions for improvement of HIVCT services. Observations were conducted by two people during two days in each facility, after authorization from its director. They were based on a checklist for the description of places, people, activities, interactions (excluding counseling sessions). Anonymity was applied in all data collection. Textual data translated into French, constituting a corpus of about 75,000 characters, were cleaned and classified into Word files (interviews) or in Dedoose^a^ (FGDs) for iterative thematic coding and content analysis.

For this article, we paid special attention to providers’ and clients’ statements and comments about the quality of care, difficulties encountered, and ethical issues. To avoid interpretation bias due to categorization, we collected and treated data on the basis of broad conceptual categories, as usual in qualitative methods [19,20]. Since ‘Individual rights’ and considerations on ‘ethics’ might not be labelled as such by respondents, especially clients, we gathered statements on attitudes and practices in HIVCT that they considered *good* or *bad*, morally acceptable or reprehensible. We compared clients' notions to WHO’s definition of ethical principles applied to HIVCT labelled ‘3Cs’ and ‘5Cs’. These data complement more general analyses of clients’ perception which have been presented elsewhere [21].

## Results

Providers who participated in individual interviews were aged 33 to 45. They included midwives (2), a nurse (1) and psychosocial counselors (3). They all had more than 5 years experience in providing HIVCT. Clients were aged 19 to 55, they included 19 women and 27 men, with a diversity in socioeconomic, occupation and education backgrounds.

The themes that emerged from an analysis of narratives and comments by providers and clients about campaigns are presented in two sections: (1) Perceptions of the campaigns and reasons for their success in attracting people for testing; (2) Perceptions about quality of care during campaigns (conditions of testing, counseling, consent, confidentiality and access) from a ‘rights’ perspective.

### Perceptions of the campaigns and reasons for their attractiveness

All providers evaluate campaigns positively, based on the large numbers of tests conducted and the high attendance at testing sites. Both providers and campaign-testers attribute this success to several factors: the availability of multiple testing sites, the fact that testing is free during campaigns (which is not always the case outside campaigns) and the information and awareness component of the national campaign. This is illustrated in the following:

‘As a counselor myself, I can give two reasons [for this success]: because testing is free and because [people] feel motivated, since during that period everybody gets information on TV and radio. They talk about AIDS everywhere.’ (Counselor)

These positive perceptions are shared by those who were tested during campaigns, those who were tested outside of campaigns, and even by non-testers:

‘Everybody knows that it is simple that way, and it is free.’ (Campaign tester). ‘And during the campaign, information is everywhere, even if you don’t want to listen, you will hear the message and it will lead you to getting tested.’ (Off-campaign tester). ‘For me, the benefit of campaigns is public information, and also if you get an HIV-positive result, they can take care of you and give you good advice.’ (Non-tester)

Beyond availability and geographical and economic accessibility of testing during campaigns, clients and providers also mention factors that seem important in their definition of quality: the convenience of testing, the ‘ripple effect’, a sense of community with other testers, the cordial atmosphere, and the anonymity of testing during campaigns.

Clients’ reports provide details on the convenience of testing during campaigns. Contrary to usual situations, they do not need to plan for testing nor spend much time to find testing facilities, as expressed in this narrative:

‘During your campaign […] I saw the parked vehicle. I asked people, and they said they were doing HIV tests. Then I decided on the spot to do it. Later, I pushed two people to do the same. After 30 minutes I got the result and saw it was negative, as did the two people I had pushed; this is what they told me. I was walking around and I did it immediately. But since I was born, I had not undergone testing; this was the first time. I was very pleased about it, and that I could know my HIV status. I really appreciated it.’ (Campaign tester)

The convenience of testing during campaigns means that the degree of motivation required to test is lower than in the usual situations when testing is based on a voluntary request. Campaigns appear as a special opportunity to test:

‘Since it’s campaign time, I told myself I should not miss this opportunity.’ (Campaign tester)

Campaigns are also perceived as having a ‘ripple effect’ when individuals are encouraged by their friends or relatives, in addition to the campaign’s information and awareness-raising aspect.

‘For instance, if we are having tea and discussing, we may talk about the campaign. We might convince one of us that he should get his test and tease him about it […] (laughs). We convince each other that we should do so and it ends with the entire group going together for an HIV test.’ (Campaign tester)

Testers’ testimonies describe diverse situations when this ‘ripple effect’ happens: people meet others on the way to the testing center and advise them to go for testing together; a person encourages her/his partner after completing his or her own test and being reassured about HIV-status; young students go together to a testing center after school; *‘some people see that the testing center is very crowded and they join’*; some people inform others that HIV testing is ‘provided for free’; a friend may convince a new tester by explaining his own testing itinerary; some people show others their HIV-negative results and this motivates them to get tested. Narratives suggest that motivations are not solely related to peer pressure nor to an active quest for diagnosis or care, but rather to a more diffuse sociability, whereby people get tested because they happen to accompany others who are going to test.

In addition to convenience and the ripple effect, some providers and clients explain the success of campaigns and the high attendance by the open and friendly atmosphere in testing sites:

*‘Everybody cannot usually go to ordinary testing sites, but here it is very different. It is in the city center, it’s open to everybody, you find many people there, everybody is chatting, and the atmosphere is easy-going. It is then easier to come for testing in this context.’* (*Campaign tester*)

Another client tested during a campaign emphasizes the impact of the open atmosphere and exchanges on people’s attitudes and considers the interview for our study as another positive example of communication:

*‘If you go to campaign sites, you will find aspects that motivate you to go forward in your life, such as this interview, for instance.’* (*Campaign tester*)

### Perceptions about quality of care during campaigns from a ‘rights’ perspective

The narratives of providers and clients about experience with testing reveal their perceptions of the quality of testing services, and of the ethical dimension of testing conditions, counseling and consent, confidentiality and accessibility.

#### Testing conditions

Besides appreciating accessibility of CT during campaigns, some people report being discouraged by the high number of clients at testing sites and prefer to come back after the campaign:

‘The problem was the overflow of people. Thus, some had to leave before getting tested because they did not have time to wait.’ (Campaign tester)

High attendance was confirmed by observation in VCT sites, particularly during the afternoon, when crowds were waiting in all available spaces, discussing, listening to information provided by a prevention worker or watching videos about HIV, some of them complaining about waiting time. Duration of waiting time for a client before getting counseling was 15 to 90 mn. The longer time spans were observed at peak hours (usually from 3:00 pm to 5:00 pm) or when a large number attended a testing site (for instance students after an information session was organised in their college).

Teams were keen on serving all clients whatever their motivation for testing, and subsequently had to manage the crowd. When the crowd was too important or at the end of the day, in some sites the staff explained to clients who had not registered yet that testing before and after campaigns is similar, and encouraged people to come back later, after the campaign. But providers did not apply triage based on matters of personal HIV risk or history of testing. Clients report that health workers messages encourage testing whatever the personal situation:

‘The message was really clear: it is good to get tested.’ (Off-campaign tester)

Clients were not discouraged by multiple testing, as shown by this statement:

*‘Personally I get tested since I was in 9*^*th*^*grade and I try to do it every 1*^*st*^*December since that day has been chosen by United Nations for fighting AIDS in the world.’ (Campaign tester)*

Observations showed that a testing itinerary entails five steps in the VCT facility: registration, participation in collective information, pre-test counseling, testing and post-test-counseling. In some facilities, testing was done by a lab attendant in a separate room whereas in others, it was performed by the counselors. To efficiently use clients’ waiting time and manage the crowd, while providing useful information, teams invited members from Community-Based Organizations specialised in HIV Information and Prevention to organize more educational sessions. In some facilities, members of People living with HIV-support associations were in charge of overseeing client reception, help clients through the different steps and encourage them to participate in information sessions rather than merely waiting.

Since each step in the itinerary presents a point at which clients may drop out, particularly if there is a long wait, provider and teams try to retain clients, intended to shorten the time clients spend on site, keep clients ‘busy’ and interested, engage them in interactions that set a continuum in care provision and a kind of relational commitment from clients. However there were some situations when clients who tested at campaign sites interrupted their itinerary before getting their test results, which happened when test results were not delivered right away, but several hours later or the day after. Such interruptions suggest that perhaps people had not fully consented in the first place. Providers mention that by setting the whole process within a single time span, rapid testing has helped reduce dropout and made greater continuity possible between a counselor and a client for pre- and post-test counseling. Clients appreciate this organization during campaigns because they do not need to repeat the same explanation during post-test counseling and because it personalizes the relationship when they meet the same counselor both times. According to providers, who had little information on this topic, the issues related to connecting clients to follow-up care services are the same during and outside campaigns.

Providers mention insufficient test kits, due to the 2008 situation when demand greatly exceeded expectations and testing sites ran out of kits. Providers had to improvise ways to procure extra test kits or reagents through unconventional channels, referring clients to other testing sites or giving them appointments the week after the campaign. Many providers worked several hours after official closing time to meet the high demand. In one site, clients complained that after getting counseling, they were asked to return the next day for testing due to closing time. However, despite the high demand, neither clients nor providers mentioned that a client was sent back without being tested as a result of crowd management in testing sites.

#### Counseling and consent

The most frequently used strategy to manage high attendance was to shorten the duration of counseling, particularly for pre-test and for post-test with HIV-negative clients. Observation confirms that the duration of pre-test counseling sessions varied from 5 to 10 mn, and post-test from 3 to 10 mn, according to the number of people waiting to be counselled. A critical issue for providers is to keep high quality counseling when it is performed in a short time span. Regarding pre-test counseling, a provider compares campaign and routine situations:

‘In daily conditions it becomes a conversation, [for] sometimes a client speaks then she discusses during 30 mn before we can do the test. But during campaigns it is difficult to give this time when others are waiting outside’. (Midwife)

Providers have several ways to optimize available time during campaigns. Since information on HIV is provided extensively by the media and through collective presentations in many sites, some providers focus on interacting with clients and establishing a dialogue on personal matters without providing general information and checking whether it is understood. Other providers mention that they strictly comply with national counseling guidelines regarding the content of counseling but encourage clients to give short answers to their questions:

‘To have enough time, you won’t let the client explain what he knows about HIV, you will only give him information’. (Counselor)

Some providers mention that clients ask fewer questions during campaigns, since many are not really at risk for HIV, unlike clients at fixed facilities who tend to experience more complicated situations, related to exposure or worry about transmission. Other providers make extra appointments for clients after the campaign to provide pre-test counseling that is ‘long enough’ to ensure that clients are ready to undergo testing.

Regarding post-test counseling, some providers report that during campaigns, they take less time than usual for HIV-negative people, and a few clients deplore the shortened contact:

‘During campaign, [...] I think, it is short… I do not consider it really as a counseling after testing’. (Campaign tester)

At the post-test stage, providers schedule extra appointments with HIV-positive clients to get them to accept their HIV result and to connect them with care.

Regarding consent, although campaigns are based on voluntary testing and all people who are tested are assumed to have consented, there are circumstances when consent may be an issue. Providers report cases of a client requesting a test for someone else, or a client requesting to test under a partner’s or relative’s pressure (a situation not specific to campaigns). Providers also mention situations when people give consent without appearing to be sufficiently informed, which happens during campaigns when they come without reluctance to a facility. The following quotes illustrate providers’ and clients’ concerns about motivation:

‘[Some people] follow the movements of others. It is ‘follow-ism’, and sometimes you may feel they are not ready. During counseling you feel that they would not be ready to accept a (HIV-positive) result’. (Midwife)

‘I usually meet young students; some of them will go for testing because their classmates have been tested, and their results were HIV-negative, thus they will go to see if they have the same result’. (Off-campaign tester)

In such cases, the campaign’s momentum may undermine individual consent. Providers report using strategies to insure that client’s psychological dispositions are optimal, such as requesting an extra counseling session before testing or making an appointment for the week following the campaign to check that the testing request is truly the individual’s choice rather than perceived obligation. In our study, no client or provider reported instances when someone regretted after having taken an HIV test during the campaign.

#### Confidentiality

Confidentiality is a key concern for providers during campaigns, regarding two matters: the patients’ private life discussed mainly during pre-test counseling and the result of the HIV test discussed during post-test counseling. Providers request that counseling sessions be conducted without observer and ask third persons to wait outside, even when invited by the client. They also try to secure spaces for counseling and testing that offer at least some privacy, even when rooms usually devoted for counseling are occupied and extra rooms must be arranged. Providers must ensure that it is impossible to hear dialogues from outside, which is difficult when there is crowd in every corner and when opening a window is the only protection against high temperatures in the Sahelian area. One provider explains that rooms used for counseling are also used for other matters, particularly in health facilities where daily care activities are maintained during campaigns. In that case, counselors must use behavioural strategies to avoid breaches in confidentiality during counseling:

‘If somebody must come in to get something here, I stop counseling, I stop and wait and I explain to the client that the person is a health worker who wants to get something. Generally it is necessary to close the door to be alone with a client’. (Midwife)

Providers also have to find a way to maintain confidentiality after post-test counseling, when the client who was announced a HIV-positive result may show emotions and non-verbal signs that reveal his/her status against his/her will to anyone in the HIVCT facility. Providers try to ensure that the path out of the room should not expose the client to public scrutiny, particularly from people waiting to be counseled who may understand the reason for client’s positive or negative feelings. Providers find it particularly difficult when groups of clients come to test together, for example students who may find out their classmates’ results based on their involuntary disclosure, as explained here:

‘Three girls came together, they were about twenty years old. Unfortunately one was HIV-positive. When I told her, she was shaking, she was crying, I had to spend some time with her to make her understand, then to get calm before getting out. Otherwise the other ones would immediately know that her results were positive’. (Counselor)

In these cases, providers’ capacity to protect confidentiality is limited to hiding all differences between reactions to HIV-positive and HIV-negative results in the post-test process. For this purpose, they control the time duration for post-test counseling, scheduling extra appointments to complete counseling instead of providing a longer session that would be a sign for an HIV-positive result. They also help the HIV-positive client hide emotions for instance through covering one’s face with a scarf or getting calm before leaving the room. Providers may also postpone post-test counseling and give another appointment at a time when there will be no risk of coming with acquaintances or meeting other clients after HIV-positive status disclosure:

‘For instance when we get results and they are HIV-positive, we may tell this person that results have not arrived yet and she should come back when other ones have left’. (Nurse)

Some providers also give advice on how to keep one’s HIV status secret and avoid unintentional disclosure by preparing ready-made answers to be used by the client towards curious people. Most providers also advise HIV-positive person to avoid intentional disclosure except for partner and one or two persons (parent or brother/sister), as part of post-counseling:

‘We try to make them understand that results should not be shared, because it is confidential’. (Counselor)

Clients generally appreciate rapid testing not only for better time management but also for reasons of confidentiality, since when a single appointment is sufficient the risk of meeting an acquaintance in the testing site is reduced. However, clients still express some concerns about confidentiality during campaigns. Some clients (mostly non-testers) mention that they fear interactions with other clients, especially when they participate in information sessions together and, subsequently, may feel pressure to share their HIV-test results with those who tested with them. This social pressure does not necessarily mean that personal results are disclosed in answer to third party queries: people disclose spontaneously because they feel relieved by their result. In certain social situations they feel that they should disclose, for instance to the person who advised them to take a test, as reported by a client:

‘I have advised two people to do testing. Both did it and came to tell me confidentially that they were HIV-negative, then I asked them if they were happy and they said they were’. (Campaign tester)

Providers also mention concerns about disclosure by those tested HIV-negative, which may indirectly reveal that someone is HIV-positive if he/she refuses to disclose:

‘Amongst the youth it is difficult. When they get out they immediately share results. Really we cannot manage that. It [the problem] is mainly with students, each one [proudly] says ‘I am HIV-negative’. (Counselor)

Some clients protect themselves from the perceived social pressure to disclose by going to a testing center alone, although they received the information on the campaign collectively, for instance at their workplace.

However, clients also speak positively about the openness they encounter in testing sites for talking about sexuality or personal risk. Clients show some ambivalence about confidentiality and secrecy at HIVCT testing sites during and off campaigns: they appreciate sharing ideas and personal matters with providers and with other people, except when it comes to an HIV-positive result.

#### Accessibility

The accessibility of counseling and testing during campaigns that results in high attendance may be valued differently by providers and clients. Providers do not express any ethical concern regarding repeat testers and they do not object to the use of testing services by those not at risk. They consider that every test is useful, no matter why it is performed. Even when testing sites are subject to heavy demand, and despite organizational challenges and heavy workloads, providers do not set up triage according to perceived risk of being HIV-positive, but rather use strategies to extend testing after the campaign so as to respond to all clients.

Both providers and clients note that the crowding that happens during campaigns brings benefits by allowing for a degree of anonymity among clients. Several providers state that many people who get tested during campaigns would not have tested individually for fear of being suspected of immoral behaviors, requesting an HIV test in VCT sites has long been a matter of stigma:

‘When they see you near VCT site they believe you have AIDS and when a week later you have malaria, that’s it [they diagnose it as AIDS]. And if you are a woman or a girl outside VCT it means that you are not serious. Or if you enter a testing site and pass by somebody you know they will say Ah, you too you want to get an HIV test, it means that you did something shameful and you want to check if you were infected’. (Nurse)

Some testers emphasize that requesting an HIV test in a crowded site is easier since no one is singled out or recognized.

The social diversity of clients during campaigns, which contrasts with other modes of testing that target particular groups, is also seen as an advantage. A provider states that men do not usually feel at ease in mother-and-child health facilities where they may be tested as partners of pregnant women in PMTCT programs. Similarly, under-18 clients who need a parent authorization usually do not dare ask for their parents’ signature; older people do not want to be seen requesting an HIV test. But individuals from these various categories do test during campaigns, taking advantage of the diversity of people in the crowd to ensure anonymity. Providers mention that they counseled many new testers who had never dared to undergo voluntary testing outside of campaigns. For the same reasons, some testers explain that they had decided or needed to do their test for a long time, but they waited for the campaign to actually take the test because they prefer to test in that context.

Some comments show that high client demand seems to achieve more than increasing uptake. Providers interpret crowds at testing sites as an accomplishment in equity, reaching those who usually have difficulty in accessing care and those who may not use health services, such as villagers or men. They emphasize the diversity of clients during campaigns:

‘We received very young students, who came to get a parental authorization form and had it signed. We also had elders, women, men…’. (Counselor)

‘Everybody cannot go to usual HIVCT sites, but then (during campaigns) it is different, testing is available in the city center, it is open to everybody…’. (Off-campaign tester)

Campaigns’ accessibility is valued in terms of good quality of care and answer to individual rights to testing.

## Discussion

This qualitative study of providers’ and clients’ perceptions of HIV testing campaigns in Burkina Faso shows very positive valuations of campaigns for their attractiveness and also for the quality of care and protection of confidentiality, counseling, consent and access to HIV-testing. This is not surprising since during campaigns, testing services work at their best, with fewer shortages in testing devices or health workers, and HIV tests are more available, accessible and acceptable than at other times. Providers, clients tested during campaigns and outside of campaigns and even clients who have never been tested all share the belief that campaigns are successful ‘*because they motivate people towards testing’*. Campaigns do not compete with other strategies but attract people to testing sites even after the campaigns themselves have ended. In general, in Burkina Faso, there are few critical voices against campaigns, and they tend to come from people who have never taken an HIV test and whose knowledge about testing practices and conditions seems out of date. This positive perception of campaigns as a public health strategy is in line with the results of the 2008 campaign, which surpassed its quantitative objectives [17], and more generally with clients’ overall favorable perceptions of HIV testing during campaigns in countries such as Kenya, Malawi, and Gabon [11,22,23].

High attendance during campaigns is not only due to their attractiveness, but also to a growing demand for HIV testing from the general population. Many clients consider that everyone is at risk of HIV transmission and that regular testing improves prevention. This perception is consistent with public preventive messages delivered by the media or in schools. It also echoes other incorrect lay perceptions about HIV transmission whereby half of the general population think that mosquitoes transmit HIV and one-third believe they can be infected when sharing a meal with a person living with HIV [18]. Moreover, high attendance at testing sites during campaigns is permitted by in-depth change in social perception of HIV/AIDS in Burkina Faso. During the 1990s and early 2000s, lay perceptions suspected that any request for a HIV-test was motivated by a breach in norms of respectability; VCT was undertaken by clients who usually came alone and avoided being recognized to avoid being considered guilty [24,25]. In our study, clients’ comments on their reasons for testing and observations in facilities show that they do not fear moral judgment nor hide their testing practices. This evolution corresponds to the decrease in collective symbolic stigma described in Burkina Faso in the late 2000s, which has been documented [26,27]. Providers mention that through high attendance campaigns certainly contributed to normalize the request for testing and made it more acceptable. Obviously clients seem to value the friendly and supportive atmosphere at the testing sites, and narratives about information exchanges show that campaigns create social spaces for the circulation of HIV information and opinions between private and public spheres in a ‘non-problematic’ mode.

However the attractiveness of campaigns results in overcrowding at testing sites that does raise ethical issues. Providers’ comments shed light on four related problems: (1) Overcrowding reduces the time available for counseling and providers must find alternative ways to engage those who need more counseling; (2) High demand does not guarantee that every client is fully aware of the consequences of testing; (3) Clients may dropout at some step in their testing itinerary which will result in missed opportunity; and (4) Confidentiality, in particular about results, may be compromised.

Narratives show how providers try to balance individual and collective interests, and their responses to the challenges show commitment to individual care. Rather than applying triage to focus on the most at risk people, or first-testers, in order to reduce the number of clients, they set up various strategies through time and resource management to answer all demands while maintaining the quality of counseling (1). They keep checking the level of informed consent beyond a simple positive answer (2). In doing so, they incorporate ethical principles in practices in a way that is finely calibrated to a specific workplace and available resources, as do health providers in other health care settings such as psychiatric care [28]. Such adaptation to context requests experience and commitment, which are not always found in campaigns evaluated in other settings: in Lesotho for instance, where large numbers of lay counselors were involved in the 2005 ‘Know Your Status’ nationwide HIV testing campaign, training and support facilities were limited, and infringements on human rights, substandard counseling and poor linkage to care were reported [29]. In Burkina Faso, national campaigns organization is a collaborative process initiated by community-based associations and counsellors are not recruited only for the campaign but provide counseling all year round. The commitment of providers shown in the study may also depend on the concerned facilities, since all of them had long term partnerships with community-based HIV/AIDS support organizations and had experienced more than 5 campaigns at the time of the study.

To avoid dropout (3), providers and teams also contextualize ethics according to local conditions regarding arrangements of testing itineraries that may be facility-specific and more or less compatible with high attendance. Collective information sessions are organized to retain clients, and also to optimize time while increasing their knowledge and sparing time for individual counseling. Ethical principles for HIVCT translated into practices for managing retention are embroiled with lay ethical considerations and attention to clients as individuals.

A key finding of the study is the ambiguous relationship between high attendance and confidentiality (4): on the one hand, crowded facilities make it difficult to secure spaces of privacy for counseling and expose clients to unintentional disclosure of HIV result; on the other hand, a crowd composed of people with various social, age and gender characteristics avoids being singled out and favors HIVCT uptake. The contextualization of ethics by providers involves adapting to local norms regarding confidentiality and disclosure, which may be more or less compatible with high attendance at testing facilities. In India, women prefer getting post-test counseling together since group counseling protects them from the authoritative presence of their husbands or mothers-in-law, which would be acceptable in their social context [30]. By contrast, in Burkina Faso counseling is provided on an individual basis even when facilities are crowded, and providers devote much attention to avoid leakage of personal information. Providers feel responsible for the protection of secrecy about their client’s status long after counseling session, and during counseling they still advise HIV-positive clients to avoid disclosing (except to partners and a limited number of persons), independently of the reduction of stigma in society [31]. Ethnographies of campaigns have highlighted variations in the social framing of secrecy and private information sharing, in households and in health settings: in Malawi and Kenya, clients explain high uptake rates in home-based testing by stating that confidentiality is better at home than when individual counseling is performed in health facilities [9,32].

Our results show how providers manage high attendance during campaigns and translate global ethics norms into meaningful practices in their local context around confidentiality, consent, counseling, and retention. Beyond adapting to local workplace and resources, they must negotiate between two sets of values: ‘medical ethics’ – which underlies global public health norms and guidelines rooted in Western culture and human rights philosophy– and ‘ethno-ethics’ – which underlies local social and cultural norms [33]. It is the case when postponing post-test counseling after the campaign in order to help HIV-positive clients escape the felt obligation of immediately disclosing results with fellow participants. Then, providers enable clients to comply with social norms of sharing personal information and comply with medical ethics in a way, that differs from those adopted in Malawi [34].

New ethical issues arise during campaigns in the realm of individual autonomy. First, in the context of ordinary sociability practices and interpersonal relationships within family, friendship and professional networks, it may be difficult for an individual to refuse to go for testing amid collective mobilization, and clients must rely on providers during pre-testing to help them postpone testing until they are ready. Experienced clients or people who have more agency use strategies to avoid social pressure, as do those who are offered home-based HIV testing in Uganda [9]. Another ethical issue has to do with disclosure. In Burkina Faso, local cultural and social norms imply that any information obtained through a relationship should be shared and disclosed to the people who helped or who shared in the process; it should also be disclosed to people whose age, gender or position in the family define them as having higher status [35]. When confidentiality issues move from the testing sites to social spaces outside, providers express their concern about the disclosure of clients’ status, but they have very limited control over this in practice and can only warn clients, or educate the youngest among them, against open disclosure. Thirdly, the local shaping of stigma is also changing: this study shows a shift towards more subtle forms of stigma, now expressed in the relief of who got an HIV-negative result, whereas HIV-positive people are unable to show publicly the same relief. This is consistent with the shift from enacted stigma to self-stigma in people living with HIV’s perceptions and experience, as documented in Burkina Faso [36].

Another emerging issue is related to the high value given by providers and clients to accessibility of testing for anyone during campaigns. The belief that everybody needs to be tested regularly is contrary to recent WHO recommendations in low-level HIV prevalence or concentrated epidemic settings, which consider that re-testing should not be recommended to HIV-negative people [37]. People with indeterminate HIV status, high-risk or HIV-positive partner, sex workers, men who have sex with men and injecting drugs users should be prioritized for regular testing and messages should be differentiated according to individual situations. At the time of the study in Burkina Faso, it was considered unethical to discourage clients from testing, whatever their history or personal risk. Providers were in line with HIVCT national guidelines and national strategy [15,16]. Our results show that this perception is also consistent with their interpretation of individual rights and their personal commitment to clients, and is turned into practices during campaigns. Responding positively to all requests for testing also meet expectations of clients – including those who were not tested. In 2014, new national guidelines that might apply WHO recommendations have not been published yet. This may reflect the difficulty in translating global norms into changes in practices at field level, in particular when new directions are contrary to previous perceptions and practices encouraging everyone to get tested. This difficulty also underlies the absence of debate at the national level regarding testing strategies among the general population in a context of decreasing resources.

Finally, our results raise questions about gaps between global ethical norms and their local interpretation. In Burkina Faso, providers value the ‘Cs’, but they articulate principles and practices in a way that differs from WHO recommendations. The main discrepancy lies in the importance they give to accessibility and attractiveness, which have an ethical value at local level, when defined by WHO as a public health strategy. This result underscores a degree of disconnect between global, compared to local valuations, which might bring concerns for the adoption of new WHO policy at local level, at least in Burkina Faso.

This study has several limitations. The first has to do with the fact that providers and clients report on their own experiences, and, in a context of very positive convergent discourse about campaigns at the local and national levels, they may underreport unfavorable comments due to social desirability bias. To protect against such bias, clients were contacted with the help of People Living with HIV-organization members who emphasized the need for data based on actual experience. Providers were asked to compare campaign settings to other testing settings, in order to encourage them to make candid observations and help researchers understand the context of their statements. When participants’ reports were compared to recorded observations of testing sites, we found them to be consistent. Thus, we do not think that social desirability bias affected these results. Another limitation is that the study did not collect sufficient information about linkage to care. The low HIV prevalence means that the likelihood of HIV-positive results and hence referral to health care service is low, and a result, we collected very limited comments and narratives on this topic. In consequence, “Connection to care, prevention and treatment” is not considered in this article.

Other limitations of this study are related to its scope and method: the limited numbers of respondents and of observed HIVCT sites, reduce the capacity of the study to bring results that might be generalized to other sites or countries. Moreover data collection did not allow us to get detailed information about clients’ characteristics. However results draw observations that may provide a basis for discussion and further consideration either for implementation or comparative enquiries about HIVCT in low HIV prevalence settings.

## Conclusion

In a low HIV-prevalence setting, providers and clients greatly appreciate national HIV-testing campaigns, including ethical aspects, mainly because they allow everybody to get tested for HIV. Their perceptions are consistent with the WHO ‘Cs’ criteria for ethical evaluation, though they do not express their concerns in the same terms. Accessibility and attractiveness seem to be the most valued qualities of campaigns for all providers and clients. The high attendance at testing sites is evaluated more ambivalently: it facilitates individual requests for testing anonymously and fosters feelings of ‘togetherness’; but high attendance generates new ethical issues. Providers have set up strategies to handle large numbers of clients without applying triage. They also try to to ensure that any request for HIV testing is the outcome of individual choice rather social norms, that counseling is adapted to each individual despite time and social constraints and that confidentiality is respected even outside testing sites. The focus on the 3-Cs, or even on the 5-Cs, does not sufficiently incorporate accessibility and factors that make testing attractive to clients.

Beyond practices at the local level, the study brings insights into the tension between global ethical norms and locally-produced ‘contextual ethics’, to be compared with further studies in different contexts. Future global recommendations for HIV testing and counseling campaigns should consider accessibility and anticipate how testing services can respond to new issues related to high demand.

## Endnote

^a^Online software for qualitative and quantitative data analysis: http://www.dedoose.com.

## Abbreviations

FGD: Focus group discussion; HIVCT: HIV counseling and testing; MATCH: Multi-country African testing and counseling for HIV; PAMAC: Programme d’Appui au Monde Associatif et Communautaire; PICT: Provider-initiated counseling and testing; PMTCT: Prevention of mother to child transmission; VCT: Voluntary counseling and testing.

## Competing interests

The authors declare that they have no competing interests.

## Authors’ contributions

AD conceived the first version of this article, participated in the study design, conducted the literature review, the data coding and analysis, and wrote all versions of the manuscript. OKZ supervised data collection and management, and participated in the writing and revision of the manuscript. JFS facilitated the data collection and participated in the writing and revision of the manuscript. CO is the Principal Investigator on the MATCH study. She oversaw the work of all authors, and participated in all aspects of reviewing the literature, writing and editing the manuscript. All authors read and approved the final manuscript.

## Pre-publication history

The pre-publication history for this paper can be accessed here:

http://www.biomedcentral.com/1472-698X/14/27/prepub
